# *tert*-Butyl 4-(1-methyl-1*H*-pyrazol-5-yl)piperidine-1-carboxyl­ate

**DOI:** 10.1107/S1600536809010332

**Published:** 2009-03-25

**Authors:** Daniel Richter, John C. Kath, Arnold L. Rheingold, Antonio DiPasquale, Alex Yanovsky

**Affiliations:** a Pfizer Global Research and Development, La Jolla Labs, 10614 Science Center Drive, San Diego, CA 92121, USA; b Department of Chemistry and Biochemistry, University of California, San Diego, 9500 Gilman Drive, La Jolla, CA 92093, USA

## Abstract

The reaction of (*E*)-*tert*-butyl 4-[3-(dimethyl­amino)acrylo­yl]piperidine-1-carboxyl­ate with methyl­hydrazine leads to the formation of the title compound, C_14_H_23_N_3_O_2_, with a 1-methyl-1*H*-pyrazol-5-yl substituent. The plane of the pyrazole ring forms a dihedral angle of 33.4 (1)° with the approximate mirror plane of the piperidine ring.

## Related literature

For the structure of a related compound with a five-membered aromatic ring bonded to a saturated six-membered ring, see: Basil *et al.* (2002 ▶).
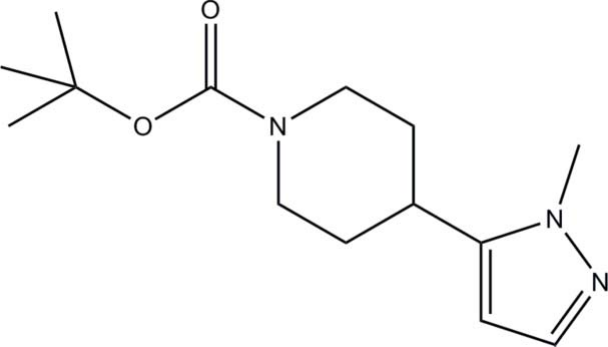


## Experimental

### 

#### Crystal data

C_14_H_23_N_3_O_2_
*M* *_r_* = 265.35
Monoclinic, 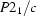

*a* = 11.356 (3) Å
*b* = 11.735 (3) Å
*c* = 11.245 (2) Å
β = 100.224 (3)°
*V* = 1474.8 (6) Å^3^
*Z* = 4
Mo *K*α radiation
μ = 0.08 mm^−1^
*T* = 198 K
0.12 × 0.12 × 0.06 mm

#### Data collection

Siemens P4 APEX CCD area-detector diffractometer
Absorption correction: multi-scan (*SADABS*; Bruker, 2001 ▶) *T*_min_ = 0.990, *T*_max_ = 0.995
14589 measured reflections
3253 independent reflections
2157 reflections with *I* > 2σ(*I*)
*R*_int_ = 0.063

#### Refinement

*R*[*F* ^2^ > 2σ(*F* ^2^)] = 0.055
*wR*(*F* ^2^) = 0.162
*S* = 1.03
3253 reflections
176 parameters
H-atom parameters constrained
Δρ_max_ = 0.28 e Å^−3^
Δρ_min_ = −0.17 e Å^−3^

### 

Data collection: *SMART* (Bruker, 1997 ▶); cell refinement: *SAINT* (Bruker, 1997 ▶); data reduction: *SAINT*; program(s) used to solve structure: *SIR2004* (Burla *et al.*, 2005 ▶); program(s) used to refine structure: *SHELXL97* (Sheldrick, 2008 ▶); molecular graphics: *ORTEP-32* (Farrugia, 1997 ▶); software used to prepare material for publication: *WinGX* (Farrugia, 1999 ▶).

## Supplementary Material

Crystal structure: contains datablocks global, I. DOI: 10.1107/S1600536809010332/rz2303sup1.cif

Structure factors: contains datablocks I. DOI: 10.1107/S1600536809010332/rz2303Isup2.hkl

Additional supplementary materials:  crystallographic information; 3D view; checkCIF report

## Figures and Tables

**Fig. 1.**
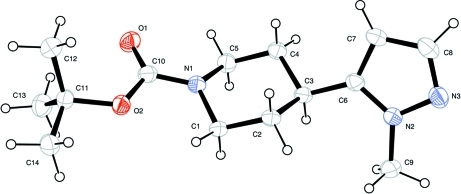
The molecular structure of the title compound showing 50% probability displacement ellipsoids and atom numbering scheme; H atoms are drawn as circles with arbitrary small radius.

## supplementary crystallographic information

### Comment

The reaction of (*E*)-*tert*-butyl 4-(3-(dimethylamino)acryloyl)piperidine-1-carboxylate with methylhydrazine leads to the formation of a pyrazole ring and can potentially produce two compounds differing in the location of the methyl group. The present X-ray study unambiguously established the structure of the product of the above cyclization as the piperidine derivative with 1-methylpyrazol-5-yl substituent (Fig.1).

The mean plane of the pyrazolyl ring forms a dihedral angle of 33.4 (1)° with the plane drawn through the C6, C3, N1, C10 atoms; this plane in fact coincides with the approximate mirror plane of the piperidine ring. The known structures featuring direct bonding between an aromatic 5-membered ring and a cycloxane/piperidine ring are surprisingly scarce. The conformation of the title compound is substantially different from that of (1*R*,2*R*,3*S*)-1-((4*S*)-4-*tert*-butyl-2-oxazolinyl)-2-phenyl-3-(phenylsulfonyl)cyclohexane (Basil *et al.*, 2002), where the 5-membered ring carries neither H atoms nor any other substituents in 1 and 3 positions and is effectively coplanar with the mirror plane of the cyclohexyl group.

### Experimental

A mixture of (*E*)-*tert*-butyl 4-(3-(dimethylamino)acryloyl)piperidine-1-carboxylate (3.95 g, 14 mmol) and methylhydrazine (0.77 ml, 1.05 eq) was refluxed for 2 h in ethanol (20 ml). The reaction mixture was then cooled down and evaporated to dryness. The residue was dissolved in 2-methyltetrahydrofurane, and the crystals formed were filtered to give 1.05 g (32%) of a white solid. It was then again recrystallized by slow evaporation of an ethylactetate solution to obtain the crystals suitable for X-ray study. ^1^H NMR (400 MHz, DMSO-d6) d, p.p.m.: 1.41 (s, 11H), 1.81 (m, 2H), 2.85 (m, 3H), 3.76 (s, 3H), 4.02 (m, 2 H), 6.05 (d, J = 2.01 Hz, 1H) 7.27 (d, J = 1.76 Hz, 1H).

### Refinement

All H atoms were placed in geometrically calculated positions (C—H = 0.95, 0.98, 0.99 and 1.00 Å for aromatic, methyl, methylene and methyne H atoms respectively) and included in the refinement in riding motion approximation. The *U*_iso_(H) were set to 1.2*U*_eq_ of the carrying atom for aromatic, methylene and methyne groups, and 1.5*U*_eq_ for methyl H atoms.

### Crystal data

**Table:**

C_14_H_23_N_3_O_2_	*F*(000) = 576
*M**_r_* = 265.35	*D*_x_ = 1.195 Mg m^−^^3^
Monoclinic, *P*2_1_/*c*	Mo *K*α radiation, λ = 0.71073 Å
Hall symbol: -P 2ybc	Cell parameters from 4631 reflections
*a* = 11.356 (3) Å	θ = 2.5–26.9°
*b* = 11.735 (3) Å	µ = 0.08 mm^−^^1^
*c* = 11.245 (2) Å	*T* = 198 K
β = 100.224 (3)°	Block, colorless
*V* = 1474.8 (6) Å^3^	0.12 × 0.12 × 0.06 mm
*Z* = 4	

### Data collection

**Table:**

Siemens P4 APEX CCD area-detector diffractometer	3253 independent reflections
Radiation source: fine-focus sealed tube	2157 reflections with *I* > 2σ(*I*)
graphite	*R*_int_ = 0.063
φ and ω scans	θ_max_ = 28.2°, θ_min_ = 1.8°
Absorption correction: multi-scan (*SADABS*; Bruker, 2001)	*h* = −14→15
*T*_min_ = 0.990, *T*_max_ = 0.995	*k* = −15→15
14589 measured reflections	*l* = −13→5

### Refinement

**Table:**

Refinement on *F*^2^	Primary atom site location: structure-invariant direct methods
Least-squares matrix: full	Secondary atom site location: difference Fourier map
*R*[*F*^2^ > 2σ(*F*^2^)] = 0.055	Hydrogen site location: inferred from neighbouring sites
*wR*(*F*^2^) = 0.162	H-atom parameters constrained
*S* = 1.03	*w* = 1/[σ^2^(*F*_o_^2^) + (0.0866*P*)^2^ + 0.0739*P*] where *P* = (*F*_o_^2^ + 2*F*_c_^2^)/3
3253 reflections	(Δ/σ)_max_ = 0.001
176 parameters	Δρ_max_ = 0.28 e Å^−^^3^
0 restraints	Δρ_min_ = −0.17 e Å^−^^3^

### Special details

**Table:**

Geometry. All e.s.d.'s (except the e.s.d. in the dihedral angle between two l.s. planes) are estimated using the full covariance matrix. The cell e.s.d.'s are taken into account individually in the estimation of e.s.d.'s in distances, angles and torsion angles; correlations between e.s.d.'s in cell parameters are only used when they are defined by crystal symmetry. An approximate (isotropic) treatment of cell e.s.d.'s is used for estimating e.s.d.'s involving l.s. planes.
Refinement. Refinement of *F*^2^ against ALL reflections. The weighted *R*-factor *wR* and goodness of fit *S* are based on *F*^2^, conventional *R*-factors *R* are based on *F*, with *F* set to zero for negative *F*^2^. The threshold expression of *F*^2^ > σ(*F*^2^) is used only for calculating *R*-factors(gt) *etc*. and is not relevant to the choice of reflections for refinement. *R*-factors based on *F*^2^ are statistically about twice as large as those based on *F*, and *R*- factors based on ALL data will be even larger.

### Fractional atomic coordinates and isotropic or equivalent isotropic displacement parameters (Å^2^)

**Table:**

	*x*	*y*	*z*	*U*_iso_*/*U*_eq_	
C1	0.47096 (16)	0.26958 (14)	0.23649 (18)	0.0443 (5)	
H1A	0.5242	0.3157	0.2974	0.053*	
H1B	0.3968	0.3136	0.2091	0.053*	
C2	0.53249 (16)	0.24635 (14)	0.12987 (18)	0.0446 (4)	
H2A	0.5568	0.3196	0.0978	0.054*	
H2B	0.4754	0.2087	0.0650	0.054*	
C3	0.64312 (15)	0.17030 (14)	0.16493 (17)	0.0415 (4)	
H3	0.7022	0.2119	0.2262	0.050*	
C4	0.60673 (17)	0.06158 (14)	0.22336 (18)	0.0460 (5)	
H4A	0.5525	0.0165	0.1623	0.055*	
H4B	0.6789	0.0150	0.2521	0.055*	
C5	0.54446 (17)	0.08740 (16)	0.32860 (19)	0.0510 (5)	
H5A	0.5171	0.0154	0.3606	0.061*	
H5B	0.6016	0.1243	0.3940	0.061*	
C6	0.70107 (14)	0.14487 (14)	0.05859 (17)	0.0420 (4)	
C7	0.68977 (17)	0.05354 (15)	−0.01942 (18)	0.0480 (5)	
H7	0.6425	−0.0129	−0.0166	0.058*	
C8	0.76220 (19)	0.07921 (17)	−0.10319 (19)	0.0558 (5)	
H8	0.7718	0.0307	−0.1684	0.067*	
C9	0.82255 (17)	0.32615 (16)	0.0698 (2)	0.0564 (5)	
H9A	0.8848	0.3545	0.0272	0.085*	
H9B	0.8564	0.3156	0.1555	0.085*	
H9C	0.7569	0.3814	0.0618	0.085*	
C10	0.34339 (16)	0.14793 (15)	0.34221 (18)	0.0454 (5)	
C11	0.15104 (16)	0.23821 (15)	0.35928 (18)	0.0463 (5)	
C12	0.07212 (18)	0.14266 (17)	0.2992 (2)	0.0583 (5)	
H12A	0.0699	0.1451	0.2117	0.088*	
H12B	−0.0091	0.1520	0.3160	0.088*	
H12C	0.1046	0.0692	0.3310	0.088*	
C13	0.16876 (19)	0.2322 (2)	0.4953 (2)	0.0613 (6)	
H13A	0.1967	0.1559	0.5222	0.092*	
H13B	0.0927	0.2480	0.5218	0.092*	
H13C	0.2284	0.2889	0.5302	0.092*	
C14	0.10122 (16)	0.35355 (16)	0.3152 (2)	0.0525 (5)	
H14A	0.1540	0.4138	0.3546	0.079*	
H14B	0.0212	0.3630	0.3351	0.079*	
H14C	0.0962	0.3585	0.2275	0.079*	
N1	0.44168 (12)	0.16258 (11)	0.29133 (15)	0.0440 (4)	
N2	0.77748 (13)	0.21780 (12)	0.01773 (15)	0.0458 (4)	
N3	0.81639 (14)	0.17886 (14)	−0.08198 (16)	0.0543 (5)	
O1	0.32504 (12)	0.06460 (12)	0.40018 (13)	0.0588 (4)	
O2	0.26749 (11)	0.23697 (10)	0.31802 (12)	0.0475 (4)	

### Atomic displacement parameters (Å^2^)

**Table:**

	*U*^11^	*U*^22^	*U*^33^	*U*^12^	*U*^13^	*U*^23^
C1	0.0469 (10)	0.0301 (9)	0.0581 (13)	−0.0007 (7)	0.0151 (8)	0.0013 (8)
C2	0.0499 (10)	0.0311 (8)	0.0540 (12)	0.0023 (7)	0.0127 (8)	0.0036 (8)
C3	0.0438 (9)	0.0338 (8)	0.0470 (12)	−0.0004 (7)	0.0082 (8)	−0.0029 (8)
C4	0.0483 (10)	0.0358 (9)	0.0546 (12)	0.0050 (7)	0.0110 (8)	0.0044 (8)
C5	0.0524 (11)	0.0447 (10)	0.0575 (13)	0.0096 (8)	0.0141 (9)	0.0113 (9)
C6	0.0416 (9)	0.0358 (9)	0.0478 (12)	0.0056 (7)	0.0060 (8)	0.0026 (8)
C7	0.0569 (11)	0.0353 (9)	0.0513 (12)	0.0047 (8)	0.0081 (9)	−0.0043 (8)
C8	0.0687 (13)	0.0481 (11)	0.0515 (13)	0.0151 (10)	0.0131 (10)	−0.0046 (9)
C9	0.0516 (11)	0.0455 (11)	0.0737 (15)	−0.0084 (8)	0.0151 (10)	−0.0055 (10)
C10	0.0502 (10)	0.0382 (10)	0.0478 (12)	−0.0027 (8)	0.0089 (8)	−0.0008 (8)
C11	0.0439 (10)	0.0493 (11)	0.0483 (12)	−0.0036 (8)	0.0150 (8)	−0.0024 (8)
C12	0.0547 (11)	0.0539 (12)	0.0677 (15)	−0.0089 (9)	0.0144 (10)	−0.0050 (10)
C13	0.0612 (13)	0.0716 (15)	0.0534 (14)	−0.0026 (10)	0.0167 (10)	−0.0002 (11)
C14	0.0455 (10)	0.0532 (12)	0.0595 (14)	0.0012 (8)	0.0117 (9)	−0.0057 (9)
N1	0.0465 (8)	0.0341 (7)	0.0533 (10)	0.0032 (6)	0.0141 (7)	0.0047 (7)
N2	0.0480 (8)	0.0383 (8)	0.0530 (10)	0.0031 (6)	0.0145 (7)	−0.0004 (7)
N3	0.0592 (10)	0.0511 (10)	0.0565 (12)	0.0107 (8)	0.0208 (8)	0.0020 (8)
O1	0.0623 (9)	0.0479 (8)	0.0700 (11)	−0.0008 (6)	0.0220 (7)	0.0149 (7)
O2	0.0457 (7)	0.0441 (7)	0.0558 (9)	0.0029 (5)	0.0174 (6)	0.0066 (6)

### Geometric parameters (Å, °)

**Table:**

C1—N1	1.463 (2)	C9—N2	1.454 (2)
C1—C2	1.516 (3)	C9—H9A	0.9800
C1—H1A	0.9900	C9—H9B	0.9800
C1—H1B	0.9900	C9—H9C	0.9800
C2—C3	1.534 (2)	C10—O1	1.214 (2)
C2—H2A	0.9900	C10—O2	1.351 (2)
C2—H2B	0.9900	C10—N1	1.353 (2)
C3—C6	1.494 (3)	C11—O2	1.477 (2)
C3—C4	1.525 (2)	C11—C13	1.508 (3)
C3—H3	1.0000	C11—C14	1.516 (3)
C4—C5	1.513 (3)	C11—C12	1.517 (3)
C4—H4A	0.9900	C12—H12A	0.9800
C4—H4B	0.9900	C12—H12B	0.9800
C5—N1	1.464 (2)	C12—H12C	0.9800
C5—H5A	0.9900	C13—H13A	0.9800
C5—H5B	0.9900	C13—H13B	0.9800
C6—N2	1.356 (2)	C13—H13C	0.9800
C6—C7	1.377 (2)	C14—H14A	0.9800
C7—C8	1.389 (3)	C14—H14B	0.9800
C7—H7	0.9500	C14—H14C	0.9800
C8—N3	1.323 (3)	N2—N3	1.355 (2)
C8—H8	0.9500		
			
N1—C1—C2	110.50 (14)	N2—C9—H9B	109.5
N1—C1—H1A	109.6	H9A—C9—H9B	109.5
C2—C1—H1A	109.6	N2—C9—H9C	109.5
N1—C1—H1B	109.6	H9A—C9—H9C	109.5
C2—C1—H1B	109.6	H9B—C9—H9C	109.5
H1A—C1—H1B	108.1	O1—C10—O2	124.54 (17)
C1—C2—C3	111.88 (15)	O1—C10—N1	124.25 (17)
C1—C2—H2A	109.2	O2—C10—N1	111.19 (15)
C3—C2—H2A	109.2	O2—C11—C13	110.63 (16)
C1—C2—H2B	109.2	O2—C11—C14	102.07 (14)
C3—C2—H2B	109.2	C13—C11—C14	110.36 (16)
H2A—C2—H2B	107.9	O2—C11—C12	110.10 (15)
C6—C3—C4	111.67 (14)	C13—C11—C12	112.29 (17)
C6—C3—C2	111.56 (15)	C14—C11—C12	110.95 (17)
C4—C3—C2	108.97 (14)	C11—C12—H12A	109.5
C6—C3—H3	108.2	C11—C12—H12B	109.5
C4—C3—H3	108.2	H12A—C12—H12B	109.5
C2—C3—H3	108.2	C11—C12—H12C	109.5
C5—C4—C3	111.68 (14)	H12A—C12—H12C	109.5
C5—C4—H4A	109.3	H12B—C12—H12C	109.5
C3—C4—H4A	109.3	C11—C13—H13A	109.5
C5—C4—H4B	109.3	C11—C13—H13B	109.5
C3—C4—H4B	109.3	H13A—C13—H13B	109.5
H4A—C4—H4B	107.9	C11—C13—H13C	109.5
N1—C5—C4	110.87 (15)	H13A—C13—H13C	109.5
N1—C5—H5A	109.5	H13B—C13—H13C	109.5
C4—C5—H5A	109.5	C11—C14—H14A	109.5
N1—C5—H5B	109.5	C11—C14—H14B	109.5
C4—C5—H5B	109.5	H14A—C14—H14B	109.5
H5A—C5—H5B	108.1	C11—C14—H14C	109.5
N2—C6—C7	105.57 (17)	H14A—C14—H14C	109.5
N2—C6—C3	122.99 (15)	H14B—C14—H14C	109.5
C7—C6—C3	131.39 (16)	C10—N1—C1	123.62 (14)
C6—C7—C8	105.30 (17)	C10—N1—C5	118.57 (15)
C6—C7—H7	127.4	C1—N1—C5	114.12 (14)
C8—C7—H7	127.4	N3—N2—C6	112.94 (15)
N3—C8—C7	112.44 (17)	N3—N2—C9	118.98 (15)
N3—C8—H8	123.8	C6—N2—C9	128.05 (17)
C7—C8—H8	123.8	C8—N3—N2	103.74 (15)
N2—C9—H9A	109.5	C10—O2—C11	121.27 (13)
